# Effects of vaccination and population structure on influenza epidemic spread in the presence of two circulating strains

**DOI:** 10.1186/1471-2458-11-S1-S8

**Published:** 2011-02-25

**Authors:** Murray E Alexander, Randy Kobes

**Affiliations:** 1 National Research Council Canada, Institute for Biodiagnostics; 435 Ellice Avenue, Winnipeg, MB, R3B 1Y6, Canada; 2 Department of Physics, University of Winnipeg, 515 Portage Avenue, Winnipeg, MB, R3B 2E9, Canada

## Abstract

**Background:**

Human influenza is characterized by seasonal epidemics, caused by rapid viral adaptation to population immunity. Vaccination against influenza must be updated annually, following surveillance of newly appearing viral strains. During an influenza season, several strains may be co-circulating, which will influence their individual evolution; furthermore, selective forces acting on the strains will be mediated by the transmission dynamics in the population. Clearly, viral evolution and public health policy are strongly interconnected. Understanding population-level dynamics of coexisting viral influenza infections, would be of great benefit in designing vaccination strategies.

**Methods:**

We use a Markov network to extend a previous homogeneous model of two co-circulating influenza viral strains by including vaccination (either prior to or during an outbreak), age structure, and heterogeneity of the contact network. We explore the effects of changes in vaccination rate, cross-immunity, and delay in appearance of the second strain, on the size and timing of infection peaks, attack rates, and disease-induced mortality rate; and compare the outcomes of the network and corresponding homogeneous models.

**Results:**

Pre-vaccination is more effective than vaccination during an outbreak, resulting in lower attack rates for the first strain but higher attack rates for the second strain, until a “threshold” vaccination level of ~30-40% is reached, after which attack rates due to both strains sharply dropped. A small increase in mortality was found for increasing pre-vaccination coverage below about 40%, due to increasing numbers of strain 2 infections. The amount of cross-immunity present determines whether a second wave of infection will occur. Some significant differences were found between the homogeneous and network models, including timing and height of peak infection(s).

**Conclusions:**

Contact and age structure significantly influence the propagation of disease in the population. The present model explores only qualitative behaviour, based on parameters derived for homogeneous influenza models, but may be used for realistic populations through statistical estimates of inter-age contact patterns. This could have significant implications for vaccination strategies in realistic models of populations in which more than one strain is circulating.

## Background

Human influenza infection is characterized by seasonal epidemics. This occurs because influenza A is able to maintain its presence in human populations by evolutionary adaptations to population-wide immunity, resulting in mutations that gradually change viral antigens allowing the virus to evade immune detection, a process known as “antigenic drift”. On account of these rapid mutations, vaccination for influenza must be updated annually on a global basis, following surveillance to monitor the appearance of new strains [1]. Antigenic drift also diminishes vaccine efficacy for mutant strains, but may still confer partial immunity to these strains. Therefore, understanding the short-term evolution of influenza virus is crucial to developing seasonal vaccines. Conversely, vaccination of a population may influence the short-term evolution of the virus, for example by decreasing the number of hosts in which the virus may replicate.

In general, during a single influenza season, more than one viral strain is circulating. It is known [2,3] that when suitable invasion conditions are satisfied, stable coexistence of two different strains is possible. The coexistence of two or more strains in a population will influence their individual evolution; and furthermore, the selective forces acting on the strains will be mediated by the transmission dynamics in the population. For example, the infection of hosts by one strain will reduce susceptibility to other strains, thereby limiting their spread in the population [4]. In addition, the time lag in emergence of a second strain following onset of an epidemic by a first strain will be influenced by the strategy and timing of vaccination [5]. It is clear that viral evolution and public health policy are strongly interconnected, and understanding the population-level dynamics of coexisting viral influenza infections, when vaccination of the population is to be undertaken, would be of great benefit in designing such vaccination strategies [6].

In [6], a homogeneous model of two viral strains was developed, incorporating cross-immunity and delay in emergence of the second strain. It was found that for small delay and large cross-immunity, infections with both strains appeared as a single epidemic wave; on the other hand, with sufficient delay, a second epidemic wave is possible. Further, for sufficient delay and high cross-immunity, the population of susceptible hosts may become so depleted as to prevent a second wave. These findings, together with possible impact of vaccination on antigenic drift, suggest that vaccination would be an important factor to include [6].

In large populations, contacts between individuals are not uniform, as assumed in the homogeneous model [6]. Typically, the number of contacts per day per individual is much smaller than the population size, and the structure of the corresponding ‘contact matrix’ plays an important role in the development of the pattern of the disease [7]. The effects of spatial correlations [8], such as occur when community structures are present [9], were illustrated in the spread of drug resistance in a network with mild clustering [10]: the spread of the resistant strain occurred more rapidly, and at significantly lower treatment levels, than was predicted by the homogeneous model.

The present paper extends the model in [6] in a number of ways. The model includes either pre-vaccination or vaccination during the epidemic, of a predetermined part of the population. The contact structure is modelled as a Markov network [11], in which the distribution of degrees of the nodes (i.e., number of contacts for individuals in the population) is specified. In addition, the model allows a distribution of ages in the population by incorporating a prescribed number of age classes. The Markov assumption for the contact network allows the specification of structural parameters such as assortativity [12] and clustering [13-15] that are important characteristics of social groupings. These generalizations enable vaccination to be targeted according to age group and ‘contact number’ (degree of node), which in general respond to the vaccine in different ways. The model inevitably contains many parameters and allows a wide range of network structures to be specified; in addition, initial conditions can be specified in many different ways. Therefore, in this paper, only a simplified network model will be investigated. The structure of the network is comprised of uncorrelated nodes, with degree distribution specified as a truncated scale-free form [7]. Furthermore, for simplicity only one or two age-classes are considered, where, for the latter, the median age is chosen to separate the two classes. While a detailed age distribution, characteristic of a real population, could be specified, the present results are intended to be illustrative only and to allow comparison with the corresponding homogeneous model. The network model can potentially be useful in describing specific populations, such as a small or large city, in which case the network structure and age distribution would need to be determined from statistical analysis of demographic and census data [16].

Section 2 describes the model in broad terms, and lists some of the parameter values used; technical details are given in the Appendix. Section 3 presents the results of simulations, in which the cross-immunity and delay in appearance of the second strain infection are varied. These results are also compared with those produced by the corresponding homogeneous model, to ascertain the importance of structure in the network for determining the time-course and final extent (“total attack rate”) of the disease. Finally, Section 4 discusses these results, some possible extensions of the model, and implications for vaccination strategies in more realistic models based on specific demographic data.

## Methods

**Figure 1:**
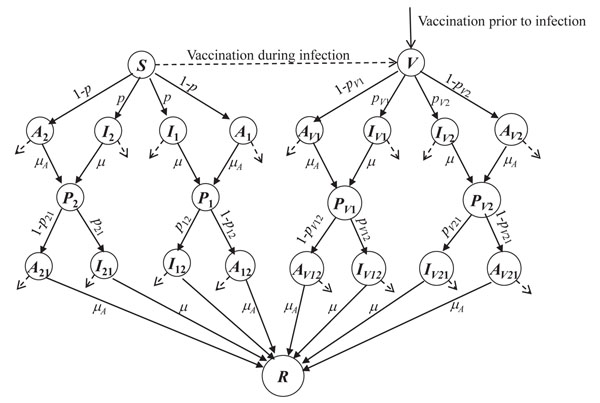
**State flow diagram for the two-strain influenza model.*** S* denotes the susceptible state, without prior vaccination. Other susceptible individuals may receive vaccination prior to the onset of infection, or after infection has appeared, and are denoted by *V*. Despite vaccination, some individuals become infected and follow a similar sequence of infection states to that of the susceptibles. States (and parameters) originating from vaccination are denoted by a subscript ‘*V’*. States representing symptomatic infection by strain *j* are denoted by *I_j_*, and correspondingly those infected asymptomatically by *A_j_.* Double-subscripted states indicate that the individual was previously infected with one strain, and is now progressing through infected states of the other strain. *P_j_* denotes individuals partially recovered from infection by strain *j*, but still susceptible to infection by the other strain. *R* denotes the class recovered from successive infection by both strains. In addition, infected individuals may die (at rates *d* or *d_A_*) and transfer (via the dashed arrows) to a disease-induced death class *D* (not shown). See text for details and explanation of the parameters.

The state flow diagram of the model is given in Figure 1, which represents either the population counts in various compartments in the homogeneous model or the state of any given node (labelled by infection state, degree- and age-class) in the network model. The model describes the evolution of two concurrent strains of influenza infection, over a duration short compared to the natural lifespan of an individual in the population, and for this reason birth and natural death processes are ignored, and furthermore the number of individuals in each age class remains constant. Since we consider a static contact network, the degree class of each individual is also fixed. Therefore, each individual in the population belongs to a unique class (*k*,*a*), where *k* denotes the number of contacts, and *a* the age class, and the total number of individuals in each (*k,a*) class is constant. *S* denotes the susceptibles and *V* denotes individuals receiving vaccination either prior to the onset of the first infection or after this onset.

Vaccination prior to the onset of infection is specified by the fraction of susceptibles in each age class receiving vaccination. For vaccination occurring during an outbreak, the following model is used: for individuals in any given (*k,a*) class, the rate of vaccination at any given time is (i) proportional to the current number of susceptibles in the class; (ii) an increasing function of the total current (symptomatic) infection in the population as a whole, saturating at a prescribed rate. This was done to attempt to model the social response to an outbreak in the population, in which the greater the number of infected individuals the more likely that susceptible individuals would avail themselves of existing vaccination opportunities. The precise mathematical specification of this response is given in the Appendix.

The baseline transmission rate of infection between a susceptible-infected pair of individuals is denoted by τ. The actual rate will depend on the age-classes that these individuals belong to, and whether the susceptible individual of the pair is seeing infection (by either strain) for the first or second time. These various possibilities are accounted for by expressing the actual transmission rate as τ times a factor, which depends on age classes involved, whether this is the first or second infection, and whether the individual has received prior vaccination. Details are given in the Appendix and in Table 1.

**Table 1:** Model parameters and their values [6].

Parameter	Value	Parameter	Value
τ	3.5 d^-1^	*p_V_*_12_	0.3
δ*_A_*	0.142	*p_V_*_21_	0.06
δ_12_= δ_21_ (δ)	0≤δ≤1	µ	0.244 d^-1^
δ_*V*1_	0.8	µ*_A_*	0.244 d^-1^
δ_*V*2_	0.9	σ_1_	0.8
*p*	0.6	σ_2_	0.4
*p*_12_, *p*_21_	0.3	*d*	0.002 d^-1^
*p*_V1_	0.12	*d_A_*	0.002 d^-1^
*p*_V2_	0.36	T*	10d, 60d

The values used in the simulations reported here correspond to those reported in [23,24] for influenza. See Section 2 and Appendix for descriptions of parameters. Although data pertain to mean field (homogeneous) models, a correspondence between these and network models of this paper - see Appendix - enables one to apply these parameters to the latter type of model. In particular, a relationship between the transmission coefficient of the mean field model, β, and the baseline transmission rate τ between individuals in contact, enables one to assign a value to τ via the mean field expression for the basic reproductive number *R*_0_, as described in the Appendix. For the simulations, with pre-vaccination rate V_0_ = 0.2, a value *R*_0_ = 1.9 was assumed, representative of seasonal influenza and close to the value *R*_0_ = 1.8 used in [25]. Without vaccination, the corresponding value of *R*_0_ would be 2.34 (see Table 5).

States labelled with *I* denote symptomatic infection, and those labelled with *A* denote asymptomatic infection. The *P* states describe immunity to one strain but not the other: *P_j_* is the state with immunity to strain *j* (*j* = 1, 2), and *R* the state with immunity to both strains. In this model, we exclude co-infection: at any given time, an individual may be infected with at most one strain. State *I_j_* denotes infection with strain *j;* and *I_jk_* denotes previous infection with (and subsequent recovery from) strain *j* and current infection with strain *k* (where *k ≠ j*)*.* A similar notation applies to the *A*-classes. The efficacy of the vaccine against strain *j* is denoted by σ*_j_*.

Subscript ‘*V*’ denotes states of infection (or partial recovery) arising from failure of the vaccine; and as before, labels states with infection due to, or partial recovery from, one of the strains. Following vaccination, infection due to strain *j* occurs with probability (1-σ*_j_*). In general, for seasonal influenza, the vaccine is targeted against the earlier-occurring strain 1 virus; its efficacy against the later-occurring strain 2 (mutated) virus is expected to be less, i.e., σ_2_ < σ_1_. As in [6], the delay T* in appearance of strain 2 in the population is a parameter of the model.

In Figure 1, the diverging pairs of directed edges are labelled with branching ratios for each strain of infection, with two pairs of such edges emanating from *S* and *V* classes. For example, if *S* is infected with one of the strains, it has a probability *p* of being symptomatically infected, and 1-*p* of being asymptomatically infected. (We assume that *p* is the same for both strains). Since *S* may be infected with either strain, there are two pairs of branches emanating from *S* in Figure 1. Similarly, there are two branch pairs for *V*, representing infection due to failure of the vaccine.

After recovery from one strain of infection, an individual is still, in general, susceptible to infection by the other strain: individuals in state *P_j_* (i.e., recovered from infection with strain *j*), can become infected with strain *k* (*≠ j*) but with diminished probability *δ_jk_.* The probability of such infection being symptomatic is denoted by *p_jk_.* Similarly, for individuals who have received prior vaccination but still become infected by strain *j*, the probability of strain *k* infection is denoted by *p_Vjk_*. Finally, the model allows for the possibility of disease-induced death, denoted by the state *D.* The rates at which these occur are assumed to be *d* or *d_A_* for symptomatic and asymptomatic infections, respectively, regardless of which of the disease states precede death; furthermore, the death rates - as with other parameters of the model – may depend on the age group in which the death occurs.

The converging directed edges in this Figure are labelled with the recovery rates from infection: either µ (symptomatic infection) or *µ_A_* (asymptomatic infection), where we assume that these rates are the same for both strains, regardless of whether this is the first or second infection for that individual. The parameter values used in the simulations are given in Table 1.

For the homogeneous model, we may apply the technique of the next-generation matrix [17] to derive the basic reproductive number *R*_0_. In general, the second strain appears after infection due to the first strain has begun, so that *R*_0_ can be calculated using a one-strain sub-model. With this assumption, we find

where *S*_0_, *V*_0_ denote the initial numbers of susceptible and vaccinated individuals, respectively, in the population, and β is the transmission coefficient. To establish a relationship between β and the baseline transmission rate τ between individuals in contact, we construct a (single-age class) network in which the ‘edge probability’ of randomly choosing an edge, one of whose vertices has degree *k*, is uniform. By relating this to the mean field model we derive (see Appendix)

where *k*_1_ = vertex degree of population sub-class into which the Strain-1 infection is introduced at time *t* = 0, and *k_max_* = maximum vertex degree in the finite network (*k_max_* = 20 in the simulations). If we choose for *V*_0_ = 0.2, a conservative value *R*_0_ = 1.9 for influenza [10], then using the above expressions for β and *R*_0_ we derive τ = 3.5 d^-1^ for the transmission rate to be used in the simulations. The value of *R*_0_ corresponding to this τ in the absence of vaccination is *R*_0_ = 2.34.

In keeping with the definition of the two age class model (see Appendix), the estimates of death rates [18,19] arising from symptomatic or asymptomatic infection (*d, d_A_*, respectively) for the two age-class model correspond to the general population above and below the median of the age distribution *P_a_* which, for the city of Vancouver, is about 38 years [20]. We assume that the death rates due to natural causes are negligible, and choose nominal values for the disease-induced rates: *d*(*a*_1_ ) *= d*(*a*_2_ ) = 0.002 d^-1^ (Ref.[10]). These rates vary with the particular circulating influenza strains. Furthermore, we set *d = d_A_* in this illustrative study.

In the model described above, the total number of individuals *N_k_*_,_*_a_* in each (*k,a*) class is fixed, and hence the total population *N* (summed over all (*k,a*) classes) is constant. Therefore, by dividing the number of individuals in class (*k,a*) in state *X* at any given time by *N*, we may express the model in terms of the probability *X_k_*_,_*_a_*(*t*) that a randomly chosen individual is in state *X*, and belongs to class (*k,a*), at time *t*. The resulting set of ordinary differential equations describing this deterministic model is given in the Appendix.

## Results

The initial state was specified as follows. For pre-vaccination, a prescribed fraction *V*_0_ (*a*) of individuals in each age class *a* receive vaccination. Infection by strain 1 is introduced into fraction *ε*_1_ of the remaining susceptibles residing in a single class (*k*_1_,*a*_1_). After the strain 1 infection has spread through the population for a time T*, a strain 2 infection is introduced into a fraction ε_2_ of class (*k*_2_,*a*_2_) individuals. In the simulations, we use ε_1_ = ε_2_ = 0.5; *k*_1_ = 5, *k*_2_ = 10, and for the two age class model, *a*_1_ = 1, *a*_2_ = 2. As previously mentioned, it is assumed that no individual may be infected with both strains simultaneously. The simulations were performed using three models: (1) network model with two age classes; (2) network model with one age class; and (3) the homogeneously-mixing 'mean field' model. For (1) and (2), the structure of the network was chosen to have a scale-free form [7], with the number of individuals (nodes of the contact network) with *k* contacts being proportional to *k*^-2.5^[21], and 1 *≤ k ≤ k_max_* = 20. Furthermore, the degrees of the nodes of the network were assumed to be uncorrelated: although real networks show significant correlation structure – e.g., clustering and associativity [12-15] - the purpose of the present simulations is to illustrate the general effects of departure from the homogeneous mixing assumption. A value *R*_0_ = 1.9 was fixed for the mean field model with *V*_0_ = 0.2.

**Figure 2:**
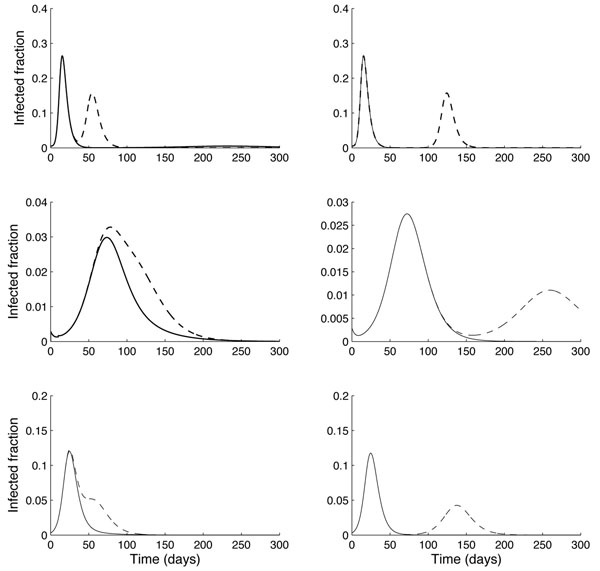
**Comparison of models: Varying cross-immunity and delay for introducing strain. 2** Comparison of results from network model for one age class (top row), two age classes (middle row), and mean field (bottom row) models. Solid curves are for δ = 0.4 (60% cross-immunity) and dashed curves for δ = 0.9 (10% cross-immunity). Plots in the left column are for the appearance of the second strain 10 days after strain-1 infection starts; right column plots for the appearance of the second strain 60 days after initial strain-1 infection. For both network models, strain 1 was introduced into class *k* = 5 and strain 2 into class *k* = 10. For the two age class model, infections were started in different age classes. Note that a second wave appears only when the cross-immunity is small (dashed curves), apart from a small, delayed outbreak in the one-age class model.

Figure 2 compares the results from the network model using one age class (top row), two age classes (middle row), and the mean field model (bottom row), for delays of T* = 10 days (left column) and 60 days (right column) for introducing strain-2 infection into a prescribed sub-population. Solid curves correspond to a large (60%) cross-immunity (with *δ* ≡ *δ*_12_ =*δ*_21_ = 0.4) between strains, and dashed curves to a low (10%) cross-immunity (*δ* = 0.9). These results show that the level of cross-immunity has a significant effect on whether a second wave appears: if it is too high (60%, δ = 0.4), then the second wave does not appear in any of the three models. For the 2-age class model (middle row), the (first) peak infection occurs at a slightly lower value for the larger cross-immunity: 0.03 vs. 0.033 fraction of the population; however, the timing of these peaks (~75 days after initial infection) is not sensitive to the level of cross-immunity. Similar conclusions apply to the one age class network model and the mean field model, though the timing of these peaks is different for different models.

As expected, if the second strain is introduced after the strain 1 infection has been largely cleared from the population (as is the case when T* = 60 days: right column), then the first and second waves behave as distinct, non-interacting one-strain epidemics. However, when T* is only 10 days (left column), there is still a significant presence of strain 1 infection in the population: the infections in the two age-class model merge into a single broad peak, whereas the other two models show two distinct peaks, with the second peak occurring in both models ~50 days after initial infection.

It is therefore apparent that the two age class network model exhibits a larger delay in peak infection – for both first and second waves – compared to the one age class and mean-field models. This can be accounted for by the reduced transmissibility between classes compared to within one class, as well as reduced transmissibility within the second age class (see the *M* matrix in the Appendix). (Recall that strain 1 and 2 infections are introduced into different age classes in the two age class network model). Such differences in delays between mean-field and structured models have been observed elsewhere [10], and underline the importance of spatial structure in determining the course of an epidemic.

**Figure 3:**
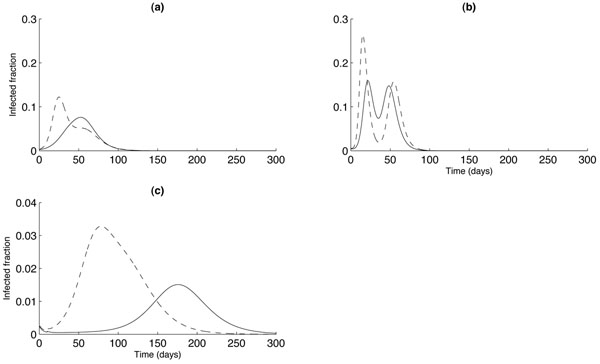
**Comparison of models: Prior-vaccination coverage**. Comparison of mean-field (a), one- and two age class network models ((b) and (c), respectively), for different prior vaccination coverage of 40% (solid curves) or 20% (dashed curves). For the two age class model, strain-1 and strain-2 infections occur in different age classes. The parameter δ = 0.9 (10% cross-immunity), and second strain infection begins at T* = 10 days after first-strain infection.

Figure 3 shows the effect of different levels of pre-vaccination (*V*_0_ = 0.2 [dashed curves] or 0.4 [solid curves]) on the infection profiles, for each of the three models, assuming a delay T* = 10 days in appearance of strain 2 infection. For all models, the peak infection is reduced by about 50%, and occurs slightly later, when increasing from 20% to 40% coverage. The peak infections in the two age class model, however, occur at significantly lower values than in the other two models: by a factor of 5 for the mean field model, and a factor of 10 for the one age class network model. Again, this may be accounted for by the reduced transmissibility between different age classes. Notice also that, as in Figure 2, the two age class model exhibits a single peak of infection for both levels of vaccination.

**Figure 4:**
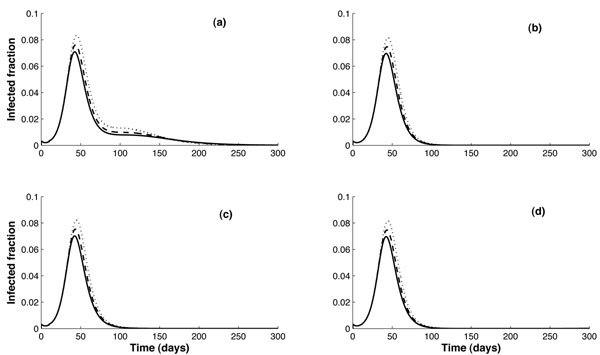
**Comparison of models: Vaccination during outbreak.** Comparison of total clinical infection resulting when vaccination is introduced during a disease outbreak, for different vaccination rates. Left column is for T* = 10 days, right column for T* = 60 days. Top row: δ = 0.4; bottom row: δ = 0.9. The underlying model is a two age class network model, and vaccination rates in each (*k,a*) class are proportional to the number of susceptible individuals in that class. These rates are determined by the total infection in the general population, and saturate at a value of ω_0_ per individual per day (see Appendix), where ω_0_ = 1 (solid curve), 0.5 (dashed curve) or 0 (dotted curve).

In Figure 4, the two age class network model is used to explore the effects of vaccination during an epidemic outbreak, with no vaccination prior to the initial appearance of strain 1 infection, where vaccination rates are determined according to the “social response” to total infection in the population, as described in Section 2 and the Appendix. The resulting infection profiles are remarkably insensitive to (i) level of cross-immunity, (ii) delay T*, and (iii) the rate of vaccination ω_0_. Apart from a temporary decrease in the rate of disappearance of the infection when T* = 10 days and δ = 0.4 (top left in Figure 4), there is only one peak of infection which occurs consistently at very similar times (50 days after strain 1 infection) and with peak magnitudes between 0.07 and 0.08 of the fraction of population. Comparing these results to the two age class model with pre-vaccination (middle row of Figure 2), it can be seen that the peak infection occurs about 20-30 days earlier for vaccination during the epidemic than when pre-vaccination is carried out.

Tables 2, 3, 4 compare the attack rates when individuals are infected by each strain separately (represented by the final values of the *P*_1_ and *P*_2_ compartments in Figure 1) and by both strains in succession (represented by the *R* compartment), for each of the three models. It is seen that in general, for given T* and δ, the attack rates predicted by the network models are quite different between the model types. However for pre-vaccination, within each model, strain 1 attack rates increase, while strain 2 attack rates decrease, with the delay T*. For vaccination during the epidemic, these trends are also seen though for strain 1 infection are less pronounced. This is reasonable, because for longer T*, the strain 2 infection draws upon a smaller pool of susceptibles (and individuals with failed vaccination) which are depleted by strain 1 infection for a longer time interval before strain 2 appears. Also, as expected, strain 2 attack rates are sensitive to the level of cross-immunity of the strains, decreasing sharply as cross-immunity increases from 10% to 60%. Variations between models are manifestations of the importance of heterogeneity of contact structure. The dependence on age distribution between network models in Tables 2, 3 is a consequence of our assumption (see matrix *M* in the Appendix) that transmissibility within age class 1 is greater than that within age class 2 or between age classes.

**Table 2:** Total attack rates for 2-age class network model

		Attack rates: Prior vaccination ***V*_0_** **= 0.2, ω_0_ = 0.0**			**Attack rates: Vaccination during epidemic: *V*_0_** **= 0, ω_0_ = 1.0**		
**δ**	T*	**Strain 1+2**	**Strain 1**	**Strain 2**	**Strain 1+2**	**Strain 1**	**Strain 2**
0.9	10	0.149929	0.229126	0.178316	0.141735	0.393720	0.083434
0.9	60	0.115638	0.279763	0.117244	0.000858	0.541612	0.000404
0.4	10	0.037663	0.338132	0.082410	0.008601	0.528141	0.010630
0.4	60	0.000463	0.398912	0.000749	0.000001	0.542568	0.000003

For the 2-age class network model, the initial strain-1 infection occurs in a fraction ε_1_ = 0.5 of individuals in class (*k*_1_,*a*_1_) = (5,1), and initial strain-2 infection, occurs at time T* = 10 or 60 days later, in a fraction ε_2_ = 0.5 of individuals in class (*k*_2_,*a*_2_) = (10,2). For these conditions, and the given degree- and age-distributions, the initial number of strain-1 and strain-2 infections (introduced at times *t* = 0 and T*, respectively) in a population of size *N =* 10,000, is 67 and 12, respectively.

**Table 3:** Total attack rates for 1-age class network model

		Attack rates: Prior vaccination *V*_0_ = 0.2, ω_0_ = 0.0			Attack rates: Vacc. during epidemic: *V*_0_ = 0, ω_0_ = 1.0		
**δ**	T*	**Strain 1+2**	**Strain-1**	**Strain-2**	**Strain 1+2**	**Strain-1**	**Strain-2**
0.9	10	0.706272	0.141649	0.098164	0.640962	0.139139	0.134720
0.9	60	0.706589	0.141371	0.098143	0.640968	0.139135	0.134721
0.4	10	0.136833	0.713552	0.027143	0.008607	0.775840	0.002266
0.4	60	0.000225	0.853501	0.000043	0.000058	0.785292	0.000015

For the 1-age class network model, fraction ε_1_ = 0.5 of degree class *k*_1_ = 5 is infected with strain-1 at time *t* = 0, and fraction ε_2_ = 0.5 of degree class *k*_2_ = 10 with strain-2 at time t = T* = 10 days or 60 days. The initial number of strain-1 and strain-2 infections (introduced at times *t =* 0 and T*, respectively) in a population of size *N =* 10,000, is 67 and 12, respectively.

**Table 4:** Total attack rates for mean field model

δ	T*	Attack rates: Prior vaccination *V*_0_ = 0.2, ω_0_ = 0.0			Attack rates: Vacc. during epidemic: *V*_0_ = 0, ω_0_ = 1.0		
		**Strain 1+2**	**Strain-1**	**Strain-2**	**Strain 1+2**	**Strain-1**	**Strain-2**
0.9	10	0.334151	0.319792	0.163241	0.289341	0.370007	0.125953
0.9	60	0.335045	0.328586	0.152525	0.290336	0.372194	0.123627
0.4	10	0.031496	0.623353	0.038615	0.007640	0.653452	0.009348
0.4	60	0.001286	0.664957	0.001463	0.000537	0.664308	0.000645

For the mean field model, the initial conditions were chosen to be compatible with those of the 1-age class model - see Appendix and Table 3. The initial number of strain-1 and strain-2 infections (introduced at times *t* = 0 and T*, respectively) in a population of size *N =* 10,000, is 67 and 12, respectively.

A question of importance to public health is the dependence of both the attack rate and death rate due to infection. The attack rates are shown in Table 5 for the two age class network model with T* = 60 days, and cross-immunity of 10% and 60%. For pre-vaccination, as expected, strain 1 attack rates decrease with increasing level of vaccination, at first slowly then dropping off sharply between *V*_0_ = 0.3 and 0.4 regardless of the level of cross-immunity. Interestingly, the maximum attack rate due to strain 2 infections is reached in this range, and drops off sharply thereafter. Thus, for the parameter values used, it appears that vaccination is most effective around these values, with diminishing returns for higher *V*_0_. Tables 6, 7 show that death rates due to pre-vaccination are lower than predicted for the entire range of vaccination rates during an epidemic; and in both cases the death rates decrease with increasing levels of vaccination. Analogous to the attack rates, there is a small increase in mortality for vaccination coverage around 20%, due to increased numbers of strain 2 infections at the expense of reduced numbers of strain 1 infections; however, the mortality rate drops sharply once the vaccination coverage exceeds about 40%.

**Table 5:** Effects of varying pre-vaccination fraction on total attack rates for 2-age class network model

*V*_0_	*R*_0_	δ=0.4			δ=0.9		
		**Strain 1+2**	**Strain 1**	**Strain 2**	**Strain 1+2**	**Strain 1**	**Strain 2**
0	2.34	0	0.661707	0	0.001156	0.660372	0.000374
0.1	2.12	0.000016	0.539788	0.000013	0.035076	0.500271	0.018405
0.2	1.90	0.000463	0.398912	0.000749	0.115638	0.279763	0.117244
0.3	1.68	0.022769	0.205177	0.088641	0.091647	0.135334	0.209307
0.4	1.46	0.007655	0.019366	0.274655	0.014051	0.014563	0.286775
0.5	1.24	0.000339	0.004577	0.051839	0.000754	0.004131	0.054892

In all cases, no vaccination occurs during the epidemic, i.e., ω_0_ = 0, and the delay in appearance of second strain infection is T* = 60 days.

**Table 6:** Effect on death rate of pre-vaccination

*V*_0_	Fraction of deaths Pre-vaccination		Effective *R*_0_
	**δ = 0.4**	**δ = 0.9**	
0	0.00542	0.00544	2.34
0.1	0.00442	0.00486	2.12
0.2	0.00328	0.00518	1.90
0.3	0.00279	0.00434	1.68
0.4	0.00254	0.00270	1.46
0.5	0.00047	0.00050	1.24

All results are for 2 age class network model, with delay in introduction of strain-2 of T* = 60 days, and cross-immunity of 60% (δ = 0.4) and 10% (δ = 0.9). The case of pre-vaccination only (ω_0_ = 0) is considered below.

**Table 7:** Effect on death rate of vaccination during epidemic

ω_0_	Fraction of deaths Vaccination during epidemic	
	**(δ = 0.4)**	**(δ = 0.9)**
0	0.00542	0.00544
0.1	0.00529	0.00530
0.2	0.00516	0.00517
0.4	0.00495	0.00496
0.8	0.00459	0.00460
1.0	0.00445	0.00446

All results are for 2 age class network model, with delay in introduction of strain-2 of T* = 60 days, and cross-immunity of 60% (δ = 0.4) and 10% (δ = 0.9). The case of vaccination only during epidemic (*V*_0_ = 0) is considered.

## Conclusions

We have considered extensions of the two viral strain mean field (homogenous) model introduced in [6], to explore the effects of both local network structure and the division of the population into different age classes. The present study used model parameter values (in particular, *R*_0_) originally estimated for mean field models; and in order to translate these to the network models derived in this paper, a correspondence was established between the mean field model and a limiting case of the network model (see Appendix). The two age class model assumed the age boundary was located at the median age (about 38 years for Vancouver), with vaccine efficacy of 80% in the lower age group and 40% in the upper age group.

Several notable features were observed when comparing the network models to the corresponding mean field case. Firstly, the amount of cross-immunity present is significant in determining whether a second wave of infection occurs. Due to a lower transmission rate between age classes and within the second age class, compared to within the first age class, infection levels were found to be significantly less for the two age class model than for either the one age class or mean field models. The infections occurred as either a single wave or as two successive waves. A second wave is more likely to occur the longer the delay in introduction of the second strain, since when this delay is short (~10 days) infections due to both strains merge into a single, broad peak. When a second wave does occur, the shapes of the two waves depend on when the second strain infection is introduced. If it occurs well after the first infection has run its course, then the two waves behave as distinct, non-interacting infections. The second infection peak is delayed, and its amplitude reduced, in the network model, compared to the mean field case. This behaviour reflects a longer propagation time in the network model, and has been qualitatively observed in other models, reinforcing the importance of including local network structure in realistic models.

As expected, the amount of cross-immunity between the two strains is important in determining the size of the second-strain outbreak. It was found that its size decreased sharply with increasing cross-immunity. As the level of vaccination increases, strain 1 attack rates decrease, with a sharp drop occurring around 30-40% pre-vaccination coverage; at the same time, strain 2 infections *increase* with increasing vaccination coverage, reaching their maximum somewhere in this range, and drop off sharply for higher coverage levels. This phenomenon is reminiscent of the development of drug resistance, where there is an optimal level of drug treatment (compare: vaccination coverage) that minimizes the overall infection [10]. This could have significant implications for vaccination strategies in realistic models of populations in which more than one strain is circulating.

It was found that increasing either pre-vaccination or vaccination during an outbreak, reduces the disease-induced mortality. Furthermore, pre-vaccination appears to be more effective than vaccination during an outbreak in reducing overall mortality, though this needs further investigation as it may depend critically on how the latter is implemented. This study considered only a simple model in which at any given time vaccination rates during an outbreak were governed by the total infection in the population at that time, and considers only vaccination of the susceptible class *S*, neglecting vaccination of other classes (e.g., *P*_1_ and *P*_2_ and asymptomatic cases).

As mentioned earlier, the particular form of the terms included in the model to incorporate local network structure and the effects of age classes was chosen for illustrative purposes. This approach, though, can be used on a specific population if sufficient data are available to determine realistic estimates of the age classes and network structure present and of the parameters of the model. The main difficulty is in determining the form of the two-point correlations between vertices of the contact network for a realistic particular population, and this must be derived indirectly from estimates of network structure extracted from the data [16]. An intermediate approach is to explore the effects of a few network structure parameters – e.g., clustering, associativity, betweenness, and centrality [7,16], obtaining expressions for the two-point probabilities defining the Markov network directly in terms of these parameters. This is currently under investigation.

## Appendix: Effects of vaccination and population structure on influenza epidemic spread in the presence of two circulating strains

The various parameters in the model (Figure 1 of main text) are defined below:

τ = baseline transmission rate between a susceptible-infected pair

*p* = probability of developing symptomatic infection with no prior exposure

*p_V_*_1_, *p_V_*_2_ = probabilities of pre-vaccinated individuals developing symptomatic infection from strains 1 and 2, respectively, with no prior exposure

*σ*_1_, *σ*_2_ = effectiveness of vaccine to strains 1 and 2, respectively

*δ_V_*_1_, *δ_V_*_2_ = reduction in transmissibility of strains 1 and 2, respectively, for vaccinated individuals

*p*_12_ = probability of developing symptomatic infection with prior exposure to strain 1

*p_V_*_12_ = probability of pre-vaccinated individuals developing symptomatic infection with prior exposure to strain 1

*p*_21_ = probability of developing symptomatic infection with prior exposure to strain 2

*p_V_*_21_ = probability of pre-vaccinated individuals developing symptomatic infection with prior exposure to strain 2

*δ_A_* = reduction in infectiousness due to asymptomatic infection

*µ, µ_A_* = recovery rates from symptomatic and asymptomatic infections

*δ*_12_, *δ*_21_ = level of cross-immunity induced by previous exposure to strain 1 and strain 2, respectively

*d, d_A_* = disease-induced death rates, assumed to be age-dependent but the same for each type of infection.

Define age classes 1 ≤ *a* ≤ *a_max_*, and network degree classes 1 ≤ *k* ≤ *k_max_*. Let

for *U* ∈ {*A*_1_, *A_V_*_1_,*I*_1_, *I_V_*_1_,*A*_21_, *A_V_*_21_,*I*_21_, *I_V_*_21_, *A*_2_, *A_V_*_2_, *I*_2_, *I_V_*_2_, *A*_12_, *A_V_*_12_, *I*_12_, *I_V_*_12_}, denote the force of infection for age-class *a* and degree-class *k*. Here, *M*(*a,a′*) denotes the relative transmission coefficient between age-groups, so that *τM*(*a,a′*) = transmission coefficient between a susceptible individual of age-class *a* in contact with an infected individual of age-class *a′*. Also, *P*(*k′,a*′|*k*,*a*) is the probability that an individual (node) of age-class *a* and degree-class *k* has a neighbour (adjacent node) of age-class *a′* and degree-class *k′*.

In the special case that the contact network is the same for all age-classes, *P*(*k′,a′|k,a*) = *P_a_*(*a′|a*)*P*(*k′|k*), where *P_a_* ( *a′|a*) denotes the probability that an age-class *a* individual has an age-class a′ neighbour. The two conditional distributions obey the conditions

In the general case, . If the node-degrees are uncorrelated, then *P*(*k′|k*) *= P_e_*(*k*′), where *P_e_*(*k*) is the edge distribution [22], defined as the probability of randomly drawing an *edge* connected to a vertex of degree *k*. It is related to *P*(*k*), the *vertex* distribution, by  Similarly, if the age distributions are uncorrelated, then *P_a_*(*a′|a*) = *P_a_*(*a′*). Thus, for uncorrelated age-structured networks, which are considered in this paper, *P*(*k′,a′|k,a*) *= P_a_*(*a′*)*P_e_*(*k′*). In the present study, the degree distribution follows a scale-free form [7]*P*(*k*) ~ *k^-^*^γ^, where we have chosen *γ =* 3.5. In this paper, we consider only one or two age classes (*a_max_* = 1, 2); more realistic models would incorporate demographic data on several ages classes (typically, *a_max_* ≥ 4), but as discussed in the main text, the model simulations are only intended to illustrate the effects of heterogeneous contact- and age-structure, in comparison with homogeneous models. For the simulations reported in the main paper, we have for the one-age class model: *P_a_*(*a*) = 1, and for the two age class model we chose *P_a_*(*a*_1_) = *P_a_*(*a*_2_) = 0.5, and

We extend the homogeneous (mean-field) model in [6] to a Markov network model with age structure, and include vaccination of strain 1 prior to onset of influenza outbreak. Furthermore, we extend this model to allow vaccination during an epidemic, by introducing a flux φ of susceptibles from the *S* classes to their corresponding *V* classes, according to the prescription:

(i) φ is a function of the total (symptomatic) infection in the population, *I_tot_* (summed over all *k* and *a*), and φ = 0 when *I_tot_* = 0;

(ii) φ is proportional to the population in class *S*(*k,a,t*);

(iii) φ eventually saturates at a maximum value as *I_tot_* increases.

A functional form that satisfies these criteria is

where ω_0_ is the (age-class dependent) saturation rate of vaccination, α is the value of *I_tot_* at half-saturation, and *n* > 0 governs the steepness of the response curve. In the simulations, α = 0.4 (i.e., half-saturation occurs when 40% of population is infected), and *n* = 2.

In order to incorporate death due to infection, we add a set of classes {*D*(*k,a,t*)} to the model, and (similar to the recovery rates) assume that death rates are either *d* (for all symptomatic infections) or *d_A_* (for all asymptomatic infections).

Based on Figure 1 (main text), this gives rise to the following set of 24×(*a_max_*×*k_max_*) equations:

where *S* ≡ *S*(*k,a,t*), etc., and the force of infection *Θ_U_* may be expressed as

where *U* ∈ {*A*_1_, *A_V_*_1_,*I*_1_, *I_V_*_1_, *A*_21_, *A_V_*_21_,*I*_21_, *I_V_*_21_*,A*_2_, *A_V_*_2_,*I*_2_, *I_V_*_2_,*A*_12_, *A_V_*_12_,*I*_12_*,I_V_*_12_}, and  is the **connectivity matrix**, defined by the contact structure of the population. Also,

where

This shows that the various *Θ*(*k,a,t*)*’s* describe the connectivity of a vertex of degree *k* and age-class *a* to all the infected adjacent vertices.

## Comparison with mean field model

In order to make this comparison, we need to obtain a “limiting” form of the network model that approximates the mean field model. This will enable us to obtain a relationship between the mean field model transmission rate β and the transmission rate τ between a susceptible-infected pair in the network. We consider a simplified network model consisting of a single age class, and an uncorrelated network (so that *P*(*k’|k*)*=P_e_*(*k’*)), and further that *P_e_* is a uniform distribution: *P_e_*(*k*) *=* 1/*k_max_*, where *k_max_* is the maximum degree in the network. With these assumptions, (A3) simplifies to

Therefore, the equation for *S*(*k,t*) becomes

where the term in parentheses is independent of *k*. Assume that *S*(*k,t*) *= P*(*k*)*S*(*t*), etc.; then this becomes

Summing over *k* from 1 to *k_max_*, we obtain

whereis the mean degree of nodes in the network.

Comparing this approximation with the Mean Field expression for *dS/dt*, suggests we make the following correspondence:

More realistically, since we introduce Strain-1 infections into the sub-population defined by (*k,a*) *=* (*k*_1_,*a*_1_), and taking account of the fact that *R*_0_ is defined in terms of the *first* generation of infection, it would be more accurate to replace by *k*_1_, so that

Using this approximate relationship enables us to compare the simulation of the behaviours of the network and mean-field models, by relating numerical values of the parameters β and τ through the simplified limiting case of a network in which the probability of drawing an *edge* at random from the network is uniform.

## Initial conditions for the mean field model

In what follows, it is assumed that the total population (including deaths) is normalized to unity, which is permissible since for this model it is constant. The initial conditions for the mean field model must be consistent with those of the network model. The analysis that follows applies to an arbitrary number of age classes and degree distributions.

In the network model, at *t* = 0 a fraction ε_1_ of Strain-1 infection is introduced into the sub-population in class (*k*_1_,*a*_1_). Therefore, we specify the initial conditions at *t* = 0 according to

where *P_ka_*_1_ ≡ *P*(*k*_1_ )*P_a_*(*a*_1_ ) represents the fraction of the total population in class (*k*_1_,*a*_1_). Similarly, at t = T* when strain 2 infections are introduced to a fraction ε_2_ of the sub-population in class (*k*_2_, *a*_2_), the corresponding mean field conditions are modified as follows:

whereare the changes in the susceptible and vaccinated sub-populations, respectively, and *P*_*ka*2_ ≡*P*(*k*_2_ )*P_a_*(*a*_2_ ) is the fraction of the total population in class (*k*_2_,*a*_2_).

In order to allow comparison between Mean Field and network models, all age-dependent parameters *δ_A_*, δ*_V_*_1_, δ_*V*2_, σ_1_, σ_2_, *µ_A_*, µ, *d_A_*, *d*, etc., in the network model are replaced by their age-distributed averages:, etc., where without risk of ambiguity we may drop the ‘*MF*’ superscript.

For the network model, for all age classes we set ε_1_ = ε_2_ = 0.5, *p* = 0.6, *V*_0_ = 0.2, σ_1_ = 0.8, σ_2_ = 0.4. For the two age-class model, we chose (*k*_1_,*a*_1_) = (5,1), (*k*_2_,*a*_2_ ) = (10,2); and for the one-age class model *k*_1_ = 5, *k*_2_ = 10. The (truncated) scale-free distribution *P*(*k*) ~ *k^-^*^3.5^ with *k_max_* = 20 yields *P_ka_*_1_ = 0.0067, *P_ka_*_2_ = 0.0012 (so that, in a population of *N* = 10,000, the number of infections is 67 and 12, respectively), where we are assuming *P_a_* to be uniformly distributed in the 2-age-class model: *P*_a_ (*a*_1_) = *P_a_*(*a*_2_ ) = 0.5.

Substituting these values into the expression for *R*_0_ (Section 2 in main paper), and using *R*_0_ = 1.9, *V*_0_ = 0.2, *k_max_* = 20, and *k*_1_ = 5, yields the values β = 0.8765, and τ = 3.5 d^-1^. For *V*_0_ = 0.4, using the same value τ = 3.5 d^-1^, the corresponding value of *R*_0_ is 2.34.

## Authors’ contributions

MEA wrote the bulk of the manuscript, and designed, implemented and simulated the network model from a homogeneous one constructed with assistance from Dr S. Moghadas. RK implemented and simulated the homogeneous model, and wrote the Conclusions. MEA wrote the Appendix.

## Competing interests

The authors declare that they have no competing interests

## References

[B1] Boni MF Vaccination and antigenic drift in influenza Vaccine 2008 26 Suppl 3 C8 C14 10.1016/j.vaccine.2008.04.011 18773534 PMC2603026

[B2] Castillo-Chavez C Hethcote HW Andreasen V Levin SA Liu WM Epidemiological models with age structure, proportionate mixing, and cross-immunity J 1989 27 233 258 10.1007/BF00290636 2746140

[B3] Dietz K Epidemiologic interference of virus populations J 1979 8 291 300 10.1007/BF00276314 501225

[B4] Andreassen V Lin J Levin SA The dynamics of cocirculating influenza strains conferring partial cross-immunity J 1997 35 825 842 10.1007/s002850050079 9269738

[B5] Bowman CS Arino J Moghadas SM Evaluation of vaccination strategies during pandemic outbreaks Math 2011 8 113 122 10.3934/mbe.2011.8.113 21361403

[B6] Moghadas SM Bowman CS Arino J Competitive interference between influenza viral strains Canadian Appl. Math. Quarterly in press

[B7] Newman MEJ The structure and function of complex networks SIAM Rev 2003 45 167 256 10.1137/S003614450342480

[B8] Keeling M The effects of local spatial structure on epidemiological invasions Proc Biol Sci 1999 266 859 867 10.1098/rspb.1999.0716 10343409 PMC1689913

[B9] Fortunato S Community detection in graphs Physics Reports 2010 486 75 174 10.1016/j.physrep.2009.11.002

[B10] Alexander ME Dietrich SM Hua Y Moghadas SM A comparative evaluation of modelling strategies for the effect of treatment and host interactions on the spread of drug resistance J 2009 259 253 263 10.1016/j.jtbi.2009.03.029 19344730 PMC7127136

[B11] Boguñá M Pastor-Satorras R Vespignani A Pastor-Satorras R, Rubi M, Diaz-Guilera A Epidemic spreading in complex networks with degree correlations Lecture Notes in Physics 2003 625 New York: Springer 127 147

[B12] Newman MEJ Assortative mixing in networks Phys. Rev. Letts 2002 89 208701 10.1103/PhysRevLett.89.208701 12443515

[B13] Park J Newman MEJ Solution for the properties of a clustered network Phys. Rev. E 2005 72 026136 10.1103/PhysRevE.72.026136 16196673

[B14] Serrano MÁ Boguñá M Clustering in complex networks. I. General formalism Phys. Rev. E 2006 74 056114 10.1103/PhysRevE.74.056114 17279975

[B15] Serrano MA Boguñá M Clustering in complex networks. II. Percolation properties Phys. Rev. E 2006 74 056115 10.1103/PhysRevE.74.056115 17279976

[B16] Kolaczyk ED Statistical Analysis of Network Data 2009 New York: Springer19658533

[B17] van den Driessche P Watmough J Reproduction numbers and sub-threshold endemic equilibria for compartmental models of disease transmission Math. Biosci 2002 180 29 48 10.1016/S0025-5564(02)00108-6 12387915

[B18] Alexander ME Bowman C Moghadas SM Summers R Gumel AB Sahai BM A vaccination model for transmission dynamics for influenza SIAM J 2004 3 503 524 10.1137/030600370

[B19] Dushoff J Mortality due to Influenza in the United States–An Annualized Regression Approach Using Multiple-Cause Mortality Data American Journal of Epidemiology 2005 163 181 187 10.1093/aje/kwj024 16319291

[B20] Data from the Canada Census, Population by Age Community Services Group 1996 Vancouver City of Vancouver. Vancouver Local Areas 1996

[B21] Pastor-Satorras R Vespignani A Epidemic spreading in scale-free networks Phys 2001 86 3200 3203 10.1103/PhysRevLett.86.3200 11290142

[B22] Weber S Porto M Generation of arbitrarily two-point-correlated random networks Phys. Rev. E 2003 76 046111 10.1103/PhysRevE.76.046111 17995064

[B23] Arino J Bowman C Moghadas SM Antiviral resistance during pandemic influenza: implications for stockpiling and drug use BMC Infect 2009 DOI:10.1186/1471-2334-9-8 19161634 10.1186/1471-2334-9-8 PMC2653495

[B24] Cauchemez S Carrat F Viboud C Valleron AJ Boelle PY A Bayesian MCMC approach to study transmission of influenza : application to household longitudinal data Stat 2004 23 3469 3487 10.1002/sim.1912 15505892

[B25] Gojovic MZ Sander B Fisman D Krahn MD Bauch CT Modelling mitigation strategies for pandemic (H1N1) CMAJ 2009 DOI:10.1503/cmaj.091641 19825923 10.1503/cmaj.091641 PMC2774362

